# SaBrcada: Survival Intervals Prediction for Breast Cancer Patients by Dimension Raising and Age Stratification

**DOI:** 10.3390/cancers15143690

**Published:** 2023-07-20

**Authors:** Shih-Huan Lin, Ching-Hsuan Chien, Kai-Po Chang, Min-Fang Lu, Yu-Ting Chen, Yen-Wei Chu

**Affiliations:** 1 Ph.D. Program in Medical Biotechnology, National Chung Hsing University, Taichung 40227, Taiwan; 2 Department of Pathology, China Medical University Hospital, Taichung 404327, Taiwan; 3 Institute of Genomics and Bioinformatics, National Chung Hsing University, Taichung 40227, Taiwan; 4 Biotechnology Center, National Chung Hsing University, Taichung 40227, Taiwan; 5 Agricultural Biotechnology Center, National Chung Hsing University, Taichung 40227, Taiwan; 6 Institute of Molecular Biology, National Chung Hsing University, Taichung 40227, Taiwan; 7 Smart Sustainable New Agriculture Research Center (SMARTer), Taichung 40227, Taiwan

**Keywords:** age stratification, breast cancer, data dimension raising, deep learning, survival analysis

## Abstract

**Simple Summary:**

Accurate prediction of the survival of bracer cancer will benefit appropriate medical decision-making and patient care. In this study, the breast cancer RNA sequencing (RNA-Seq) data in The Cancer Genome Atlas (TCGA) was first normalized to transcripts per million (TPM). After dimension raising, the differential gene expression data were used for different deep learning architectures testing. Among them, GoogLeNet was selected to build the survival prediction model, SaBrcada. Considering the influence of age on prognosis, it was found that adding stratified random sampling by the patient’s age of 61 could improve the accuracy of SaBrcada up to 0.798. In addition, a website tool of the same name, SaBrcada, was established to provide five kinds of predicted survival periods for clinicians to refer to.

**Abstract:**

(1) Background: Breast cancer is the second leading cause of cancer death among women. The accurate prediction of survival intervals will help physicians make informed decisions about treatment strategies or the use of palliative care. (2) Methods: Gene expression is predictive and correlates to patient prognosis. To establish a reliable prediction tool, we collected a total of 1187 RNA-seq data points from breast cancer patients (median age 58 years) in Fragments Per Kilobase Million (FPKM) format from the TCGA database. Among them, we selected 144 patients with date of death information to establish the SaBrcada-AD dataset. We first normalized the SaBrcada-AD dataset to TPM to build the survival prediction model SaBrcada. After normalization and dimension raising, we used the differential gene expression data to test eight different deep learning architectures. Considering the effect of age on prognosis, we also performed a stratified random sampling test on all ages between the lower and upper quartiles of patient age, 48 and 69 years; (3) Results: Stratifying by age 61, the performance of SaBrcada built by GoogLeNet was improved to a highest accuracy of 0.798. We also built a free website tool to provide five predicted survival periods: within six months, six months to one year, one to three years, three to five years, or over five years, for clinician reference. (4) Conclusions: We built the prediction model, SaBrcada, and the website tool of the same name for breast cancer survival analysis. Through these models and tools, clinicians will be provided with survival interval information as a basis for formulating precision medicine.

## 1. Introduction

Breast cancer is the most common cancer in women [1]. In 2020, approximately 2.3 million female breast cancer patients were diagnosed, accounting for 11.7% of new cancer cases. Breast cancer has not only become the main cause of global cancer but is also the fifth leading cause of cancer deaths worldwide, accounting for 1 in 6 cancer deaths [2,3]. To make matters worse, it has been predicted that the worldwide incidence of breast cancer is rising and that approximately 3.2 million new cases of female breast cancer will be diagnosed per year by 2050. These numbers indicate the urgent need for prevention and treatment strategies for breast cancer. Breast cancer commonly occurs in ducts or lobules. In addition to invading the original organs (breasts), malignant breast cancer has the ability to metastasize to distant organs such as bones, lungs, liver, and brain [4], which can lead to disease progression and eventually death in severe cases. Therefore, researchers continue to search for breakthroughs in the diagnosis, treatment, and palliative care of breast cancer. Especially in palliative care, reliable and accurate prognostic prediction plays a key role in decision-making regarding medical strategies [5].

Medical treatments should be decided based on the patient’s goals and expected survival time, the potential benefits and risks of treatment, and the effects on quality of life. Therefore, a comprehensive consideration of these factors determines treatment choices [6]. To predict patient survival time, many features, including pathogenesis, gene mutation, gene expression, clinical data, treatment, and general health, are typically considered for prognostic predictions [7,8]. Therefore, multiple predictors will be used in the model design and data analysis to determine the important features of the prognostic model. To date, researchers have proposed different combinations of predictors for survival analysis, death probability scoring, or when developing prediction tools or analysis platforms for prognosis. These tools are often called prognostic models, predictive models, or risk scores [9,10,11,12,13,14,15,16]. Increasing the accuracy of these prognostic models or risk scores can help patients make medical treatment decisions and provide more reliable survival analyses. In the postgenomic era, significant features are not limited to clinical information; the gene expression profiles of patients are also a crucial factor affecting prognosis [17,18,19].

To analyze gene expression, protein-coding RNAs (mRNAs) and noncoding RNAs, including long noncoding RNAs (lncRNAs), snRNAs, rRNAs, tRNAs, and microRNAs (miRNAs), were considered candidates [20,21,22,23]. With the launch of the Human Genome Project [24] and the advancement of next-generation sequencing technologies, more high-throughput RNA-seq data from cancer patients has become available for bioinformatics analyses [25]. However, the analysis of such large datasets has often previously been limited by hardware capabilities [26]. With advancements in hardware and the development of deep learning architectures, more studies have applied deep learning from the information domain to bioinformatics [27]. Chaudhary et al. first built a deep learning-based, survival-sensitive model using multiple-omics data, including RNA-Seq, miRNA sequencing (miRNA-Seq), and methylation data of liver cancer from TCGA [28]. This multi-omics model provided some valuable subtype-associated biomarkers but achieved survival subtype prediction accuracy with a C-index of 0.68 [28]. Ching et al. developed an ANN framework, Cox-nnet, to predict patient prognosis using 10 TCGA RNA-Seq datasets, including 10 cancer types. Cox-nnet achieved survival prediction accuracy with a C-index of about 0.685 and functional biological insights by automatically discovering biological features at both the pathway and gene levels [29]. Katzmae et al. used a Cox proportional hazards deep neural network to build a personalized treatment recommendation system, DeepSurv [30]. DeepSurv can employ several types of data, such as clinical records or high-throughput data, to predict survival and provide individual treatment recommendations. Based on gene and protein expression profiles of the Molecular Taxonomy of Breast Cancer International Consortium (METABRIC), it achieved survival prediction accuracy with a C-index of 0.673~0.679 [30]. Overall, compared with the complexity and diversity of genomic features, the number of samples from cancer patients from which RNA-seq data are available is limited. When the number of features is larger than the number of samples, model overfitting tends to occur, which reduces the accuracy of prediction in test data [31]. In addition, the limited availability of clinical data also affects the effectiveness of deep learning. The hospital’s inability to actively track patients leads to a loss of follow-up and censored death times for some patients. This incomplete clinical information may be the main limitation of cancer prognosis prediction [11].

The TCGA-BRCA database, most commonly used for bioinformatics prediction of breast cancer survival, records common event dates as the last follow-up date rather than the date of patient death. This may be the key factor affecting the accuracy of previous studies [28,29]. We therefore tested whether excluding such data could improve the accuracy of the prediction models. In addition, TCGA-BRCA provides the RNA-Seq data in fragments per kilobase per million (FPKM) for paired-end RNA-seq experiments only. As third-generation sequencing technologies have developed, such as single-molecule real-time sequencing (SMRT) and Oxford Nanopore’s technology, a widely applicable normalization method for different sequencing platforms is needed for survival analysis model construction. Transcripts per million (TPM) represent the relative expression level of a transcript, and the sum of all TPM values is a million in all samples. In principle, the TPM values of each transcript between samples are comparable. Thus, we normalized the gene expression data from FPKM into TPM in this study. Considering the correlation among gene expression levels, Convolutional Neural Network (CNN) learning was selected for model construction. In order to process the data for CNN learning, we used a dimension-raising strategy to raise the gene expression data into a matrix and then subtracted the data in pairs to generate a differential gene expression image (survival analysis image) and investigate the contribution of different architectures to the model’s performance. As patient age was reported to be an important feature affecting survival time [3], we also tested the effectiveness of the age stratification strategy. We also established a free website tool to provide five types of predicted survival intervals for clinicians’ reference.

## 2. Materials and Methods

### 2.1. Modeling Process

**Figure 1:**
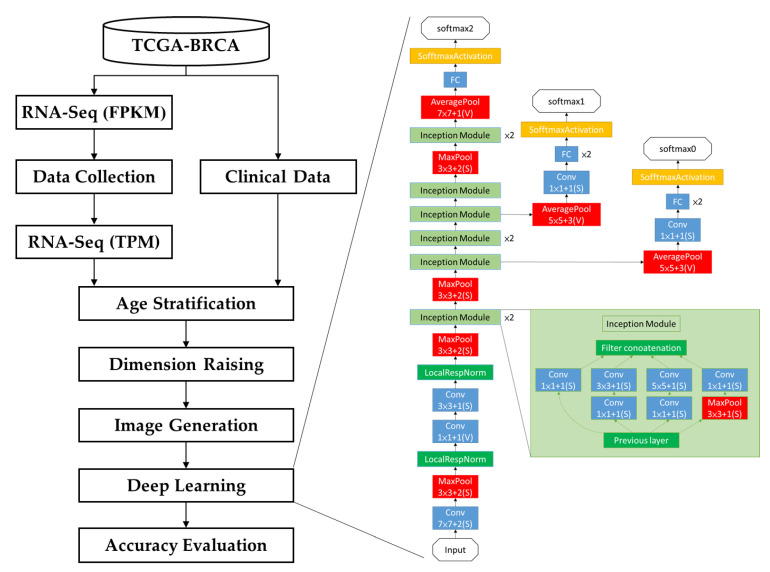
SaBrcada modeling process. RNA-seq and clinical data of breast cancer patients downloaded from TCGA-BRCA have first been filtered to exclude records with incomplete RNA-Seq expression data or missing clinical data, death dates, or age information. After converting the RNA-seq data into TPM format, it was split into two subsets based on the age of 61, and 70% of the data in each subset were used for training. Through dimension-raising, survival analysis images were generated and used for deep learning modeling. Finally, the remaining 30% of the data was used as test data to verify the accuracy of the model.

The SaBrcada modeling process is shown in Figure 1. First, we downloaded the RNA-seq data in FPKM format and clinical data of patients diagnosed with breast cancer from TCGA-BRCA and then excluded records with incomplete RNA-Seq expression data or without recorded clinical data, date of death, or age. The remaining RNA-seq data were converted to TPM format. Based on age stratification, we further divided the collected data into two datasets based on an average age of 61. Seventy percent of the patient data in the two datasets were set aside to fit the survival model. To assess the goodness of fit of the survival model by its accuracy, survival analysis images were generated following dimension raising. The two datasets from the survival analysis images were combined as the training set for model building by deep learning architectures. The remaining 30% of the patient data was collected and processed using the same procedures used to generate the test set to assess model performance.

### 2.2. Data Preprocessing

In this study, we used TCGA version 27 data. RNA-seq data from breast cancer patients were collected in FPKM format. It was noted that the original counts (reads) may be different from the true values due to the sampling environment, experimental methods, or length of each RNA [32]. Although gene length was considered, FPKM uses pair-end reads as the unit, i.e., fragments, not full transcripts. On the other hand, TPM reports the relative expression level of each transcript and formulates an identical number of total transcripts in the sample, so that gene expression levels between samples can be compared. Therefore, we chose the TPM format for further study. In addition, we also collected clinical data, including information on patient age, survival time, and race, from TCGA.

**Figure 2:**
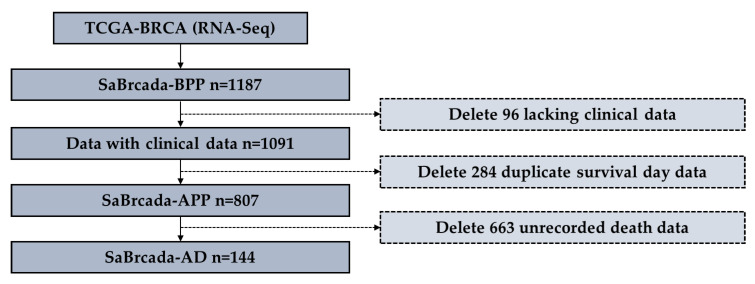
Data screening flowchart. The flowchart details how much data were deleted at each stage and why. From TCGA, 1187 samples were downloaded to construct SaBrcada-BPP before preprocessing. After excluding 96 samples lacking clinical data and further excluding 284 samples with the same survival time as other samples, we then built the SaBrcada-APP dataset, which contained 807 breast cancer cases after preprocessing. Finally, 663 samples without a death date were removed, and we obtained 144 samples with an actual death date to build the SaBrcada-AD dataset.

Before preprocessing, we downloaded a total of 1187 RNA-seq data to build the SaBrcada-BPP dataset. After excluding 96 samples with missing clinical data, we obtained 1091 data records containing clinical data. We further excluded the samples that recorded the same survival time and obtained a SaBrcada-APP dataset with 807 breast cancer cases after preprocessing. To ensure that all the samples included actual survival times, we selected 144 RNA-seq data points with actual date of death information to establish the SaBrcada-AD dataset (Figure 2). Furthermore, SaBrcada-AD was classified with stratified random sampling: the samples with a patient age younger than or equal to 61 years were included in the SaBrcada-AYT61 dataset, and the remaining samples were included in the SaBrcada-AOT61 dataset, including the samples with a patient age older than 61 years. The dataset SaBrcada-train was created by combining the two training sets of SaBrcada-AYT61 and SaBrcada-AOT61. SaBrcada-test was created by combining the testing sets of these two datasets. All 7 datasets used in this study provide information on age, survival time, and race (Table 1).

### 2.3. Age Stratification

To ensure that there was enough data in the two datasets after stratification, quantiles $Q_{1}$ to $Q_{3}$, that is, patients aged 48 to 69 years, were used as the basis for sorting the SaBrcada-AD dataset. After stratification, 70% of the patient data were extracted with the shuffle algorithm in the Random package of Python for use as the training set for the generation of survival analysis images. The other survival analysis images generated by the remaining 30% of the data were used to determine the most suitable age for stratification by accuracy evaluation. For example, there were 49 cases younger than or equal to 61 years old, which generated 2,352 survival analysis images as the training set. The other 20 cases generated 380 survival analysis images as the test set. For patients older than 61 years old, 53 cases generated 2756 survival analysis images as the training set, and 22 cases generated 462 survival analysis images as the test set.

### 2.4. Data Generation

Considering the reliability and comparability between different patients, we first normalized the 60,483 gene expression data from FPKM into TPM, and consistently arranged the expression data of genes in the order provided by TCGA-BRCA RNA-Seq for comparison of the differential gene expression between patients. According to our previous study, the difference between genes calculated using subtraction improved the predictive model of survival analysis, especially its sensitivity, compared with using traditional fold change [33]. It might be that the fold change of gene expression overestimates the effect of gene expression differences that do not reach the activation threshold [34] but underestimates the effect of gene expression differences at high expression levels; thus, subtraction was chosen. We also sorted the TPM format data according to the patient’s survival time and then subtracted the data in pairs to generate two survival analysis data types: $T_{LS}\;$(positive) and $T_{SL\;}$(negative). $T_{LS}$ is the dataset containing the data with shorter survival time subtracted from the data with longer survival time to represent the differential gene expression pattern of longer survival. In contrast, $T_{SL}$ is the dataset representing a shorter survival time. They use the same data but in reverse order to generate the data, so the total number of $T_{LS}$ is equal to the $T_{SL}$, and the balance between both positive and negative datasets is guaranteed.

**Figure 3:**
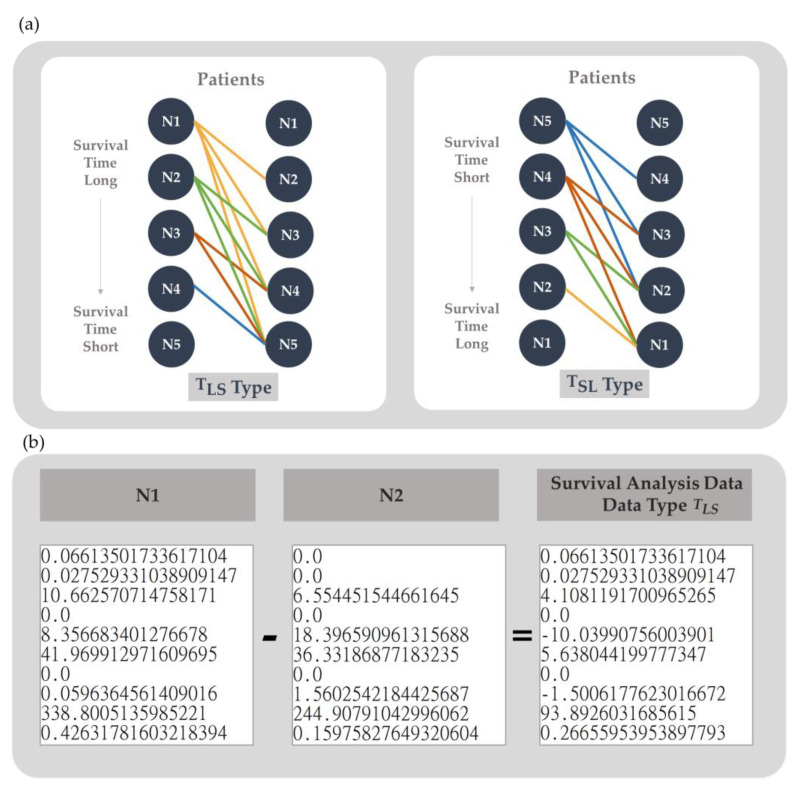
Survival analysis data generation. (**a**) The survival analysis data generation method. $T_{LS}$ (positive) is the data type that was generated by subtracting the TPM data of patients with shorter survival times from that of patients with longer survival times. $T_{SL}$ (negative) was generated by subtracting the TPM data of patients with longer survival times from that of patients with shorter survival times. (**b**) Schematic diagram of survival analysis data example. N1 and N2 indicate the gene expression of patients N1 and N2 in TPM format, respectively. The data type $T_{LS}$ is the survival analysis data generated by subtracting the TPM data of patient N2 from that of patient N1.

Taking five patients as an example, the data were arranged by the length of survival time from long to short, as N1 to N5, as shown in Figure 3a. The data type $T_{LS}$ is generated by subtracting the TPM data of N2, N3, N4, and N5 from that of N1 and then subtracting the TPM data of the remaining 3 samples from N2. It will generate n (n − 1)/2 survival analysis data, as seen in Figure 3b. In contrast, data type $T_{SL}$ is generated by subtracting the TPM data of N1, N2, N3, and N4 from that of N5, then subtracting the TPM data of the remaining 3 samples from N4, and so on, as shown in Figure 3a.

### 2.5. Data Dimension Augmentation

**Figure 4:**
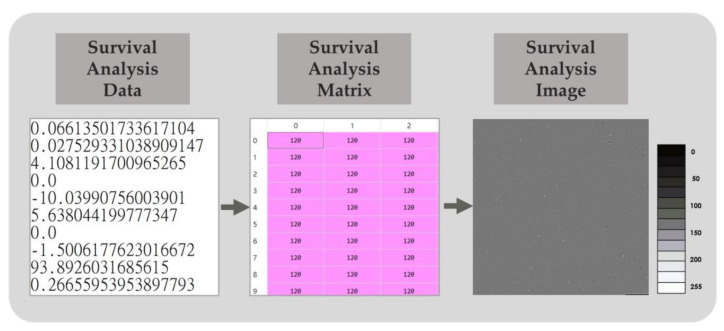
Schematic diagram of survival analysis images. By dimension raising and scaling the survival analysis data in the range from 0 to 255, a survival analysis matrix was generated for further survival analysis image conversion.

We obtained 60,483 gene expression data from TCGA-BRCA; this RNA-seq data produces a lot of features that are difficult to directly process by machine learning. CNNs designed to process data with a lot of features are therefore considered. However, CNN is more suitable for processing two-dimensional data with spatial structure. Therefore, the survival analysis data were arranged into a 246 × 246 matrix by dimension, rising from one dimension to two dimensions. Here, the 246 × 246 matrix is the smallest square matrix that can contain 60,483 differential gene expressions. All 60,483 differential gene expression levels were filled in order from left to right and top to bottom and then converted into grayscale pixel values ranging from 0 to 255. Zero represented the maximum negative difference in gene expression, and 255 represented the maximum positive difference. After filling the remaining 33 positions with 0, the survival analysis matrix was generated and finally saved in PNG file format to serve as the survival analysis images. The process is shown in Figure 4.

### 2.6. Deep Learning

We used CNN, one of the most common deep learning network architectures, implemented in PyTorch 1.9 to build a neural network framework and combined it with a Quadro GV100 32G graphics card (GPU) for model construction. The convolutional and pooling layers in the neural network architecture improve the recognition of pattern identity and the relationship between adjacent data and can learn features independently. Based on these characteristics, we used a CNN to learn features from survival analysis images. The deep learning frameworks, containing 3 inceptions and 22 convolutional layers, were used to learn different features and make comprehensive judgments on all features. For hyperparameter selection, we tested three different sets of hyperparameters by using Adam (the optimizer), Cross Entropy (the loss function), and a dropout value of 0.4.

In this study, we tested eight different deep learning architectures to build a survival prediction model. In brief, GoogLeNet, introduced by Google in 2015 [35], employs the Inception module to increase the model’s width while reducing the number of parameters. This design choice effectively addresses challenges such as overfitting, gradient vanishing, and the increased computational complexity associated with deeper architectures and a larger number of parameters. ResNet, published in 2016 [36], tackles the issue of gradient vanishing in CNNs by utilizing building blocks. This approach enables the construction of deep networks without sacrificing performance. Four of these architectures were tested in this study: ResNet18, ResNet50, ResNet101, and ResNet152. Their networks differ in depth, especially in the number of convolutional layers, which are 18, 50, 101, and 152 layers in ResNet18, ResNet50, ResNet101, and ResNet152, respectively. DenseNet, introduced in 2017 [37], optimized ResNet by establishing direct connections between each layer and all subsequent layers. This dense connectivity fosters feature reuse, reduces parameters, improves gradient vanishing, and enhances information propagation. Two of these architectures were tested in this study: DenseNet121 and DenseNet161. Similarly, the number in the architecture’s name indicates the number of convolutional layers it contains. ResNeXt, published in 2017 [38], extends the ResNet architecture by incorporating building blocks and the Inception module from GoogLeNet. This integration enhances the model’s performance, resulting in improved capabilities and efficiency.

### 2.7. Assessment of Model Performance

Accuracy is a common method to evaluate the prediction model [39]. Accuracy is calculated using Equation (1). Where *TP*, or true positive, is the number of positive predictions; *TN*, or true negative, is the number of negative predictions, and *P* and *N* are the numbers of positive and negative predictions, respectively. The accuracy ranges between 0 and 1.0. An accuracy of 0.5 represents a random prediction, and a value of 1.0 indicates that the prediction was completely consistent with the actual value.

(1) $$\mathrm{Accuracy}=\frac{TP+TN}{P+N}$$

## 3. Results

### 3.1. Survival Analysis Image Applicability Analysis

**Figure 5:**
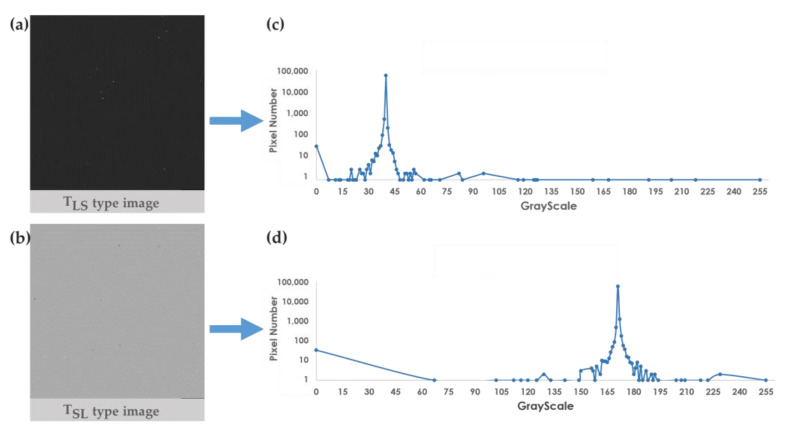
Pixel distribution diagram after image generation. (**a**) $T_{LS}$ type image; (**b**) pixel value distribution of $T_{LS}$ type image; (**c**) $T_{SL}$ type image; (**d**) pixel value distribution of $T_{SL}$ type image.

As shown in Figure 5a,b, it is difficult for the naked eye to identify the features in the survival analysis images, $T_{LS}$ and $T_{SL}$. The corresponding grayscale distributions of the survival analysis images are significantly different, as shown in Figure 5c,d. In the example shown in Figure 5, the grayscales of most pixels in $T_{LS}$ are between 30 and 45, while the grayscales of $T_{SL}$ are mostly between 160 and 180. These two types of images display sufficient differences to be learned from their features by a convolutional neural network for further survival interval analysis.

### 3.2. Deep Learning Architecture Test

Based on various features, different learning methods were selected for model construction. To detect differences between the $T_{LS}$ and $T_{SL}$ images, we adopted a deep learning method and used the SaBrcada-AD dataset for architecture testing, in which 70% of the data were used as the training set and 30% of the data were used as the test set. To identify the most suitable deep learning architecture, a total of 8 deep learning architectures, Resnet18, Resnet50, Resnet101, Resnet152, ResNext101, GoogLeNet, DenseNet121, DenseNet161, and 3 different hyperparameter combinations, Epoch 50 Batch size 8, Epoch 100 Batch size 16, and Epoch 150 Batch size 32, were tested (Table 2). Among them, we found that the most suitable architecture was GoogLeNet with a hyperparameter combination of a batch size of 32 and 150 epochs, which had the highest accuracy value of 0.6 calculated by equation 1. Therefore, SaBrcada uses this condition for model construction.

According to the results shown in Table 2, we found that GoogLeNet with a hyperparameter combination of a batch size of 32 and 150 epochs achieves the highest accuracy value of 0.6. This may be because GoogLeNet’s Inception module increases the model’s width to better extract information from differential gene expression data while avoiding excessive information gathering from higher layers. Therefore, SaBrcada uses this condition for model construction.

### 3.3. Stratification by Age

**Figure 6:**
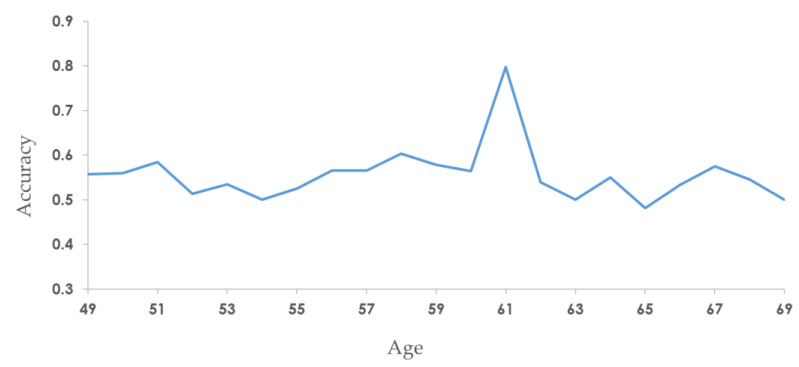
Performance of stratified random sampling by age. The X axis is the age cutoff, and the Y axis is the accuracy.

According to the clinical data of breast cancer patients, the survival time of young patients is shorter, and the survival interval of older patients is generally longer [3], which indicates that the survival days will be affected by age. For this reason, we incorporated the age feature into the model to improve accuracy by using age-stratified random sampling from quartiles $Q_{1}$ and $Q_{3}$. That is, every age between the ages of 48 and 69 is considered a cutoff for stratification and accuracy testing (Figure 6). The results showed that the highest accuracy of 0.798 can be obtained by taking the age of 61 as the cut-off for stratification, which is in agreement with the median age at the time of breast cancer diagnosis reported by the American Cancer Society [40]. Thus, SaBrcada used 61 years old as the cut-off for stratified random sampling to establish a model for subsequent survival analysis.

### 3.4. Comparison of the Previous Studies

Table 3 shows the comparison of the models constructed in this study with those of previous studies, including the accuracy, data distribution, data types, and training models. First, the SaBrcada-APP data were used to generate the survival analysis image dataset SaBrcada-APP-I for SaBrcada-APP-M model construction. SaBrcada-APP-M resulted in an accuracy of 0.5. The survival dates of most patients in the SaBrcada-APP dataset are the dates of their last follow-up days rather than the date of death. To improve accuracy, the records with a date of death were selected from SaBrcada-APP to build the SaBrcada-AD dataset. The SaBrcada-AD data were used to generate the survival analysis image dataset SaBrcada-AD-I for SaBrcada-AD-M model construction, and an accuracy of 0.6 was obtained. Grouped by age stratification, SaBrcada-AD was divided into two datasets. The dataset SaBrcada-ASYT61 included data from patients younger than or equal to 61 years, and the dataset SaBrcada-ASOT61 included data from patients older than 61 years. The data of SaBrcada-ASYT61 and SaBrcada-ASOT61 were used to generate separate survival analysis image datasets, SaBrcada-ASYT61-I and SaBrcada-ASOT61-I, for the model building of SaBrcada-ASYT61-M and SaBrcada-ASOT61-M, respectively. Model accuracy was assessed, resulting in accuracy values of 0.5 and 0.681, respectively. We used stratified random sampling to build the SaBrcada model by using the survival analysis images and the SaBrcada-I dataset. To make the SaBrcada model applicable to patients of all ages for survival analysis, the training sets of SaBrcada-I was integrated with the training set of SaBrcada-ASYT61-I and SaBrcada-ASOT61-I for modeling. On the other hand, the integration of SaBrcada-ASYT61-I and SaBrcada-ASOT61-I was used as the test set for SaBrcada-I. According to the above condition, SaBrcada achieved an accuracy of 0.798, which is better than SALMON [16], ConcatAE [41], and VAECox architecture [17]. Zhang et al. used the SALMON architecture and combined breast cancer patient data, gene set enrichment analysis, and age characteristics to construct a survival analysis prediction model with an accuracy of 0.7 [16]. ConcatAE integrated DNA methylation and miRNA expression data using principal component analysis features to develop a breast cancer overall survival prediction model with an accuracy of 0.641  ±  0.031 [41]. The VAECox framework was established based on the common features of multiple cancers to conduct transfer learning. The average accuracy of survival analysis for 10 cancers was 0.649, and the accuracy of prediction for breast cancer was also lower than 0.7 [17].

### 3.5. Assessment of the Accuracy of SaBrcada

**Figure 7:**
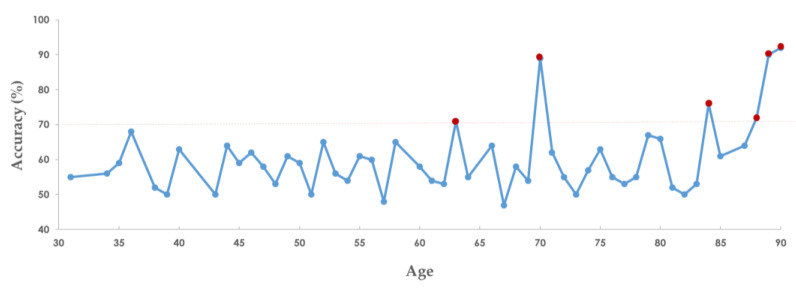
Prediction accuracy for breast cancer patients using SaBrcada by age. The *X*-axis is the age of the patient, and the *Y*-axis is the accuracy of the survival prediction. The red dots indicate that the accuracy is greater than 0.7.

In order to provide potential survival intervals via the SaBrcada web tool, suitable patients need to be identified as reference points for comparison based on their accuracy. After testing the accuracy of SaBrcada’s prediction for all patients with different ages in the SaBrcada-AD database, we found that the accuracy was higher than 0.85 for patient ages of 70, 89, and 90 years. Among them, the best performance was an accuracy of 0.92 for the age of 90 years. Patient ages of 63, 84, and 88 years also obtained accuracy values higher than 0.7, with significant differences (Figure 7).

### 3.6. Website Tools

**Figure 8:**
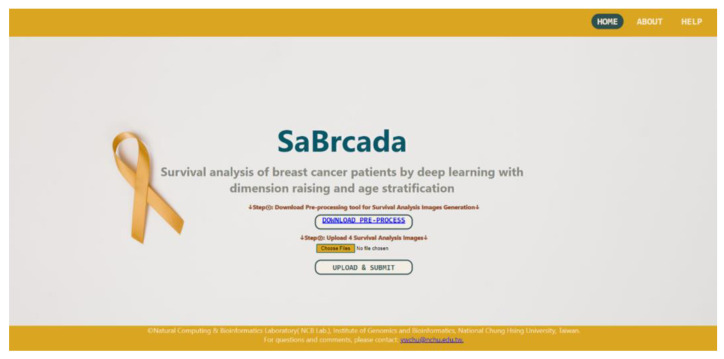
SaBrcada website tool interface. The tool is freely available at http://ncblab.nchu.edu.tw/SaBrcada (accessed on 19 July 2023). It provides a tool for generating survival analysis images and an online analysis of survival time. The outcome of the analysis is the patient’s predicted survival time, which can be classified as less than six months, six months to one year, one to three years, three to five years, or more than five years.

The purpose of developing the SaBrcada tool is to provide users with guidelines for the analysis of the survival time of breast cancer patients. Combining survival analysis and clinical experience may help clinicians choose the most suitable treatment strategies to improve the quality of life of patients. Based on the results shown in Figure 7, we identified suitable patients with higher prediction accuracy and then selected them as the reference points to compare with new data input into the SaBrcada web tool to provide potential survival intervals. The SaBrcada website interface is shown in Figure 8. SaBrcada website tool interface. The tool is freely available at http://ncblab.nchu.edu.tw/SaBrcada (accessed on 19 July 2023). It provides a tool for generating survival analysis images and an online analysis of survival time. The outcome of the analysis is the patient’s predicted survival time, which can be classified as less than six months, six months to one year, one to three years, three to five years, or more than five years. The website is freely available at http://ncblab.nchu.edu.tw/SaBrcada (accessed on 19 July 2023). SaBrcada provides preprocessing tools to transfer TPM-formatted RNA-Seq data for survival analysis image generation. After analyzing the survival analysis images uploaded by the user, SaBrcada provides the analysis information for the patient’s survival period. SaBrcada obtains two modules: the first is survival analysis image creation, and the second is survival period analysis. For survival analysis image creation, the user first downloads the preprocessing program packaged by pyinstaller and then inputs the user’s TPM file with.TXT into the corresponding file according to the age of the patient. The tool compares the input data from the user with that from four default reference patients to generate four survival analysis data. The survival analysis data are then raised to a two-dimensional matrix, and four survival analysis images are generated using the png package provided by Python. For survival period analysis, the user needs to upload the four survival analysis images generated by the preprocessing for survival analysis using the established SaBrcada model and then obtain the results. The analysis results will show the predicted patient survival period, with possible values of less than six months, six months to one year, one year to three years, three to five years, or more than five years, as a reference for clinicians to implement treatment strategies.

## 4. Discussion

### 4.1. Comparison with Past Research Models

In this study, SaBrcada, a CNN-based breast cancer survival analysis prediction model, was established by using RNA-seq data. In brief, the SaBrcada-AD dataset was selected from TCGA-BRCA based on the completeness of RNA-seq and clinical data. The RNA-seq data in SaBrcada-AD were then converted into a TPM data type to represent the relative transcript levels and used to generate the survival analysis image dataset SaBrcada-I after stratified random sampling by age of 61, which is used to build a survival prediction model based on CNN learning. By using the SaBrcada-I dataset and GoogLeNet, SaBrcada achieved the best performance of all examined frameworks with an accuracy of 0.798.

In the past, breast cancer survival analysis models typically used deep learning to extract the nonlinear characteristics of RNA-seq data and then predicted linear Cox regression survival times [16,17]. Recently, researchers began to directly use the fully connected neural network as a survival analysis model [41], and we used a similar strategy with SaBrcada. However, we made some improvements and greatly increased its accuracy. The major difference between SaBrcada and other models is that the one-dimensional RNA-seq is augmented into a two-dimensional survival analysis image, which is beneficial to the feature learning by CNN. The second is, GoogLeNet was used for construction of the prediction model instead of the Cox method. In addition, the SaBrcada website’s prediction tool provides information on the survival interval for clinicians to refer to determine treatment strategies.

### 4.2. Advantages of SaBrcada

The advantage of Sabraca is that it considers three key points that have been overlooked by previous studies on prediction model building. The first is the accuracy of the data collection. Usually, all TCGA data are used directly [28,29,30]. Thus, the last date of follow-up might be used to impute the time of death in TCGA, which may not be accurate. Whether the record contains the actual time of death may have a great impact on the accuracy of the model’s construction. Therefore, we specifically excluded the records missing the date of death to build the SaBrcada-AD dataset as the basis for SaBrcada modeling. The second point is the normalization of the data. The RNA-seq data are available in various formats. Among them, the number of reads, accounting for raw readings, may be influenced by the experimental design. FPKM counts the relative fragments per kilobase of transcript, but the total number of normalized reads in each sample may vary. Both may distort the comparison of gene expression between the two patients. On the other hand, the gene length and the sequencing depth are both normalized for TPM calculation. Consequently, the sum of all TPMs in each sample is the same. The use of TPM, a technique based on more sophisticated bioinformatics, may improve the performance of survival prediction based on gene expression. Thus, SaBrcada converts the FPKM data provided in TCGA into a normalized TPM data type to accurately present the relative expression of each gene in different patients. The third point is the impact of age on the survival risk of patients. It was reported that breast cancer patients younger than 45 years old had a worse prognosis and a shorter overall survival time than older ones [3]. Young breast cancer patients usually have multiple gene mutations involved in tumor development and cancer cell metastasis, resulting in a high cancer cell metastasis rate and a lower survival rate [42]. More than 70% of breast cancer patients over 45 years old were diagnosed with luminal A and luminal B subtypes and had the best prognosis [42]. Thus, age should be considered in prognosis predictions to reflect its impact on death risk. Consequently, SaBrcada uses age to perform stratified random sampling of the dataset to assess the effect of different age stratification cut-offs and to improve the accuracy of the model. The predictive accuracy indicated that 61 years of age is the best criterion for stratification by age, which echoes the median age of breast cancer patients reported by the American Cancer Society’s Breast Cancer Statistics Report 2017–2018 [40].

### 4.3. Directions for Future Research 

In the past, doctors analyzed the prognosis of patients by using their clinical experience, inevitably causing inconsistencies in accuracy due to individual differences. In the postgenomic era, precision medicine has become a trend. In this study, we combined gene expression and clinical data to establish a reliable survival analysis model, SaBrcada. To enrich the biological information provided, we will integrate characteristics, genetic variants [43], and coexpression network analyses. Based on this improvement, we may extract the determining factors from the black box of the survival analysis tool. This may provide a reliable prediction of survival intervals and an explainable result, including molecular information for clinicians’ reference to determine the treatment strategy for individual patients.

## 5. Conclusions

In this study, we have established a breast cancer survival analysis prediction model, SaBrcada, and its website, http://ncblab.nchu.edu.tw/SaBrcada (accessed on 19 July 2023). We downloaded the gene expression and clinical data from TCGA-BRCA. After normalization of the RNA-seq data into TPM format and dimension raising, survival analysis images were generated by differential gene expression and then subjected to deep learning architecture testing. According to their performance, GoogLeNet was selected to build the survival prediction model, SaBrcada. We also evaluated key factors affecting the performance of the survival prediction model. After filtering out the incomplete data, the performance of SaBrcada-AD-M improved to an accuracy of 0.6. Coupled with the stratified random sampling by patients’ age of 61, the performance of SaBrcada achieved an accuracy of 0.798. These results suggest that the accuracy of date of death recording and stratified random sampling by the medium age of patients will improve the performance of the survival prediction model. We hope this highly reliable survival analysis model and website tool provide information on survival interval periods for clinicians’ reference in precision medicine.

## Figures and Tables

**Table 1:** List of the datasets used in this study.

Dataset	No.	Age at Index, Median (Range)	Survival Day, Median (Range)	Race No. (%) (W, BAA, A, AIAN, NR) *
SaBrcada-BPP ^a^	1187	58 (26, 90)	912 (−7, 8605)	753 (68%), 182 (16%), 61 (5%), 1 (0.09%), 94 (9%)
SaBrcada-APP ^b^	807	57 (26, 90)	1026 (0, 8605)	583 (72%), 141 (17%), 34 (4%), 1 (0.1%), 48 (5%)
SaBrcada-AD ^c^	144	58 (31, 90)	1163 (0, 7455)	106 (74%), 30 (21%), 2 (1%), 0 (0%), 6 (4%)
SaBrcada-AYT61 ^d^	69	46 (31, 60)	1439 (227, 7455)	51 (74%), 15 (22%), 1 (1%), 0 (0%), 2 (3%)
SaBrcada-AOT61 ^e^	75	69 (61, 90)	1004 (0, 4267)	55 (73%), 15 (18%), 1 (3%), 0 (0%), 4 (5%)
SaBrcada-train ^f^	103	58 (31, 90)	1032 (0, 7455)	77 (74%), 19 (18%), 2 (2%), 0 (0%), 5 (7%)
SaBrcada-test ^g^	41	58 (31, 85)	1692 (158, 3926)	29 (71%), 11 (27%), 0 (0%), 0 (0%), 1 (2%)

^a^ Before preprocessing, incomplete data were included; ^b^ after preprocessing; ^c^ all data with an actual death interval were recorded; ^d^ Patient’s age younger than or equal to 61 years old; ^e^ Patient’s age older than 61 years old; ^f^ Combination of AYT61 and AOT61 training sets; ^g^ Combination of AYT61 and AOT61 testing sets. * W—White; BAA—Black or African American; A—Asian; AIAN—American Indian or Alaska Native; NR—Not Reported.

**Table 2:** Comparison among different Convolutional Neural Network architectures.

Architecture	Accuracy	Batch Size	Epoch
Resnet18	0.50	8	50
	0.49	16	100
	0.50	32	150
Resnet50	0.50	8	50
	0.50	16	100
	0.50	32	150
Resnet101	0.50	8	50
	0.50	16	100
	0.50	32	150
Resnet152	0.50	8	50
	0.49	16	100
	0.50	32	150
ResNext101	0.50	8	50
	0.50	16	100
	0.50	32	150
GoogLeNet *	0.55	8	50
	0.50	16	100
	0.60	32	150
DenseNet121	0.55	8	50
	0.54	16	100
	0.54	32	150
DenseNet161	0.55	8	50
	0.55	16	100
	0.53	32	150

Optimizer: Adam; Loss Function: CrossEntropyLoss. * SaBrcada adopted the architecture of GoogLeNet with Epoch 150 and Batch size 32.

**Table 3:** Comparison of SaBrcada with other breast cancer survival analyses.

Model	Number of Cancer Type	Type of Data	Patient Number	Method	C-Index * /Accuracy ^†^
SaBrcada-APP-M	1 ^a^	mRNA	807 ^c^	GoogLeNet	0.500 ^†^
SaBrcada-AD-M	1 ^a^	mRNA	144 ^c^	GoogLeNet	0.600 ^†^
SaBrcada-ASYT61-M	1 ^a^	mRNA	84 ^c^	GoogLeNet	0.500 ^†^
SaBrcada-ASOT61-M	1 ^a^	mRNA	60 ^c^	GoogLeNet	0.681 ^†^
SaBrcada	1 ^a^	mRNA	144 ^c^	GoogLeNet	0.798 ^†^
VAECox (2019)	10 ^b^	mRNA	6127 ^d^	VAE, Cox	0.649 *
SALMON (2020)	1 ^a^	mRNA, miRNA–target interactions	626 ^c^	Cox	0.700 *
ConcatAE (2020)	1 ^a^	DNA methylation, miRNA	1060 ^e^	ConcatAE	0.641 *

^a^ Only one cancer type, breast cancer; ^b^ 10 cancer types; ^c^ 70% for training, 30% for testing; ^d^ 80% for training, 20% for testing; ^e^ 60% for training, 15% for validation, 25% for testing; * the performance was evaluated by C-index; **^†^** the performance was verified by accuracy.

## Data Availability

The datasets for this study can be found in the TCGA-BRCA https://portal.gdc.cancer.gov/projects/TCGA-BRCA (accessed on 31 October 2020).
